# Difference in expression patterns of placental cholesterol transporters, ABCA1 and SR-BI, in Meishan and Yorkshire pigs with different placental efficiency

**DOI:** 10.1038/srep20503

**Published:** 2016-02-08

**Authors:** Linjun Hong, Xiangdong Xu, Ji Huang, Minggang Lei, Dequan Xu, Shuhong Zhao, Mei Yu

**Affiliations:** 1 Key Lab of Agricultural Animal Genetics, Breeding and Reproduction of Ministry of Education, College of Animal Science and Technology, the Cooperative Innovation Center for Sustainable Pig Production, Huazhong Agricultural University, Wuhan 430070, China

## Abstract

Cholesterol is a key cell membrane component and precursor of steroid hormones. The maternal cholesterol is an important exogenous cholesterol source for the developing embryos and its transportation is mediated by ABCA1 and SR-BI. Here we reported that during the peri-implantation period in pigs, ABCA1 was expressed by uterine luminal epithelium (LE) and interestingly, its expression was more abundantly in LE on mesometrial side of uterus. However, SR-BI was expressed primarily by LE, glandular epithelial cells (GE) and trophoblast cells (Tr). During the placentation period, the expression levels of ABCA1 and SR-BI proteins at epithelial bilayer and placental areolae were significantly higher in Chinese Meishan pigs compared to Yorkshire pigs. Consisitently, mRNA levels of HMGCR, the rate-limiting enzyme for cholesterol synthesis, were significantly higher in Meishan placentas than in Yorkshire placentas. Our findings revealed the routes of transplacental cholesterol transport mediated by ABCA1 and SR-BI in pigs and indicated that ABCA1 related pathway may participate in anchoring the conceptus to the mesometrial side of uterus. Additionally, an ABCA1 dependent compensatory mechanism related to the placental efficiency in response to the smaller placenta size in Meishan pigs was suggested.

The efficiency of maternal-fetal nutrients transport is closely correlated with the rate of embryo implantation and the degree of fetal development^1^,^2^,^3^. During the early implantation period (about on Days 4–15 of pregnancy in pigs), the developing porcine conceptus (including embryo, trophectoderm and associated extra-embryonic membranes) undergoes dramatic morphological changes (from spherical to tubular to filamentous forms) and migrates freely throughout the entire uterine lumen^4^,^5^. Thus the requirement for nutrients is mainly dependent on the histotroph secreted by the uterus^5^,^6^,^7^. During Days 15–20 of pregnancy, pig placentation is initiated by which the Tr starts to attach to the uterine LE to form the epithelial bilayer. The non-invasive epitheliochorial placenta is completely established around Days 26–30 of pregnancy^8^,^9^ and then the adhered trophoblast-endometrial epithelial bilayer (composed by Tr and endometrial LE) starts to develop the microscopic folded structure as gestation advanced to term^10^,^11^,^12^.

Compared to commercial western pig breeds, the Chinese Meishan pigs exhibit not only the higher litter size but also an increased physiological maturity which is correlated with the piglet survival rate before and at birth^13^,^14^. Previous studies have demonstrated that Meishan piglets at birth have increased body lipids, plasma cortisol levels and liver maturity^14^,^15^. Further investigations illustrated that the difference in degree of the piglet development before and at birth between Meishan and western breeds is mainly due to the difference in placental efficiency. Meishan pigs have ability to develop greater density of placental blood vessels as well as increased complexity of the placental folded bilayer at later stage of pregnancy^16^,^17^. It has been recognized that the efficiency of maternal-fetal nutrient transfer in pigs depends on not only the size, structure and blood flow but also the transporter abundance of placenta^18^,^19^. Thus, despite the morphologically different features, the difference in nutrient specific transporter dependent mechanism related to placental efficiency between the two breeds warrants investigation.

As the principal sterol in animal cells, cholesterol serves as the precursor of steroid hormones, including estrogens^20^. Due to the fact that the estrogen released from the conceptuses is the pregnancy recognition signal in pigs, the pig conceptus requires large amounts of cholesterol for steroid hormone synthesis during the peri-implantation period^21^,^22^. On the other hand, cholesterol is a critical structural component of animal cell membranes and functions in stabilization of the membrane surface and lipid-rich microdomains where most membrane-associated signaling reactions take place^23^. Furthermore, cholesterol is required for the synthesis of sonic Hedgehog (SHH) proteins, a secreted molecule necessary for organogenesis^24^. Thus, both the conceptus at rapidly trophoblastic elongation stage and the growing fetus at later stage of pregnancy need a large amount of cholesterol for cell differentiation and proliferation. Therefore, cholesterol is critical for successful embryo implantation and fetal development in pigs.

Although the fetus itself could synthesize the cholesterol by *de novo* synthesis, convincing evidences showed that the fetus has exogenous source of cholesterol, specifically derived from maternal circulation^25^,^26^,^27^. Up to now, researchers proposed three different mechanisms for cholesterol transport: 1) aqueous diffusion, 2) ATP binding cassette transporter A1 (ABCA1) mediated unidirectional efflux, 3) and scavenger receptor class-B type I (SR-BI) mediated bidirectional flux^28^,^29^. Although, the way of aqueous diffusion can occur with all cell types, it is inefficient. Efflux of cholesterol is accelerated when ABCA1 and SR-BI are present in the cell plasma membrane^28^. ABCA1, a member of the ABC transporter superfamily, is a membrane spanning protein that performs cholesterol and phospholipids unidirectional efflux to lipid-poor apolipoprotein A-I (apoA-I) to form precursor of high density lipoprotein (HDL)^30^. Some studies demonstrated that ABCA1 is highly expressed in rodent and human placenta and confirmed that it mediates the transport of cholesterol from mother to fetus through placenta^31^,^32^. Researchers also reported that dysfunction of ABCA1 in mice revealed dramatic pathologic changes during pregnancy, such as fetal growth restriction and increased neonatal death^33^. The scavenger receptor class B member I (SR-BI), a receptor for high-density lipoprotein, primarily mediates the bidirectional exchange of cholesterol: selective cholesteryl ester uptake and the cellular cholesterol efflux^34^,^35^,^36^. In humans, SR-BI is highly expressed in the human placental cells, allowing the growing fetus to obtain a proper portion of cholesterol from maternal circulation^37^,^38^. In mice, SR-BI is dynamically expressed in the maternal-fetal interface during pregnancy and was demonstrated to facilitate maternal-fetal cholesterol transport^39^,^40^.

Taken together, ABCA1 and SR-BI have been identified as key molecules in facilitating transport of cholesterol in mammalian placentas. However, only the expression of SR-BI mRNA was detected in pig placentas by RT-PCR^41^. Therefore, to understand the mechanism of cholesterol secretion and transport in the uterus and placenta in pigs, we evaluated 1) cell-specific localization and expression level of ABCA1 and SR-BI in uterus and placenta during pregnancy; 2) comparison the expression patterns of ABCA1 and SR-BI in uterus and placenta in Yorkshire and Meishan pigs.

## Results

### Expression patterns of ABCA1 and SR-BI proteins in Yorkshire and Meishan pigs uterus and conceptus during peri-implantation period (Days 12,15 and 18 of pregnancy)

**Figure 1:**
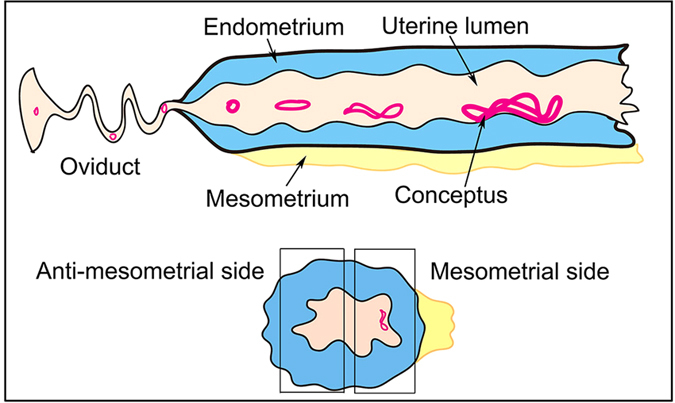
Schematic diagram shows that the attachment site of pig conceptus locates at the mesometrial side of uterine horn.

**Figure 2:**
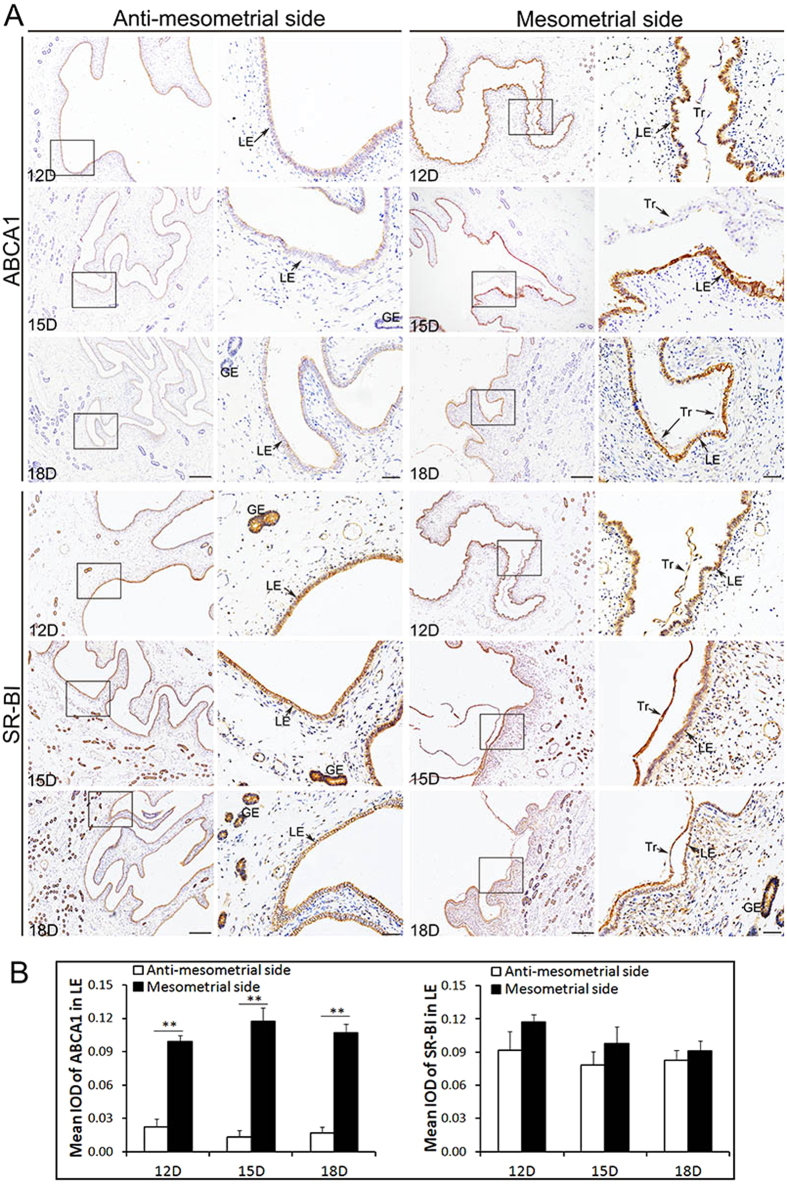
Immunohistochemical analysis of ABCA1 and SR-BI at the maternal-fetal interface (including the anti-mesometrial side and mesometrial side of the uterus) during the peri-implantation period in Yorkshire pigs. The sections stained with isotype matched normal rabbit IgG served as negative control (the control images of ABCA1 and SR-BI were in the Figs 5 or 7). (**A**) Images stained with ABCA1 and SR-BI antibodies on Days 12, 15 and 18 of pregnency in Yorkshire pigs. The results show that the ABCA1 signals were observed mainly to LE at the mesometrial side and were barely detected in LE at the anti-mesometrial side. The positive signals were also barely detected in Tr and GE. The SR-BI signals were observed in LE, GE and Tr. The stain intensity of SR-BI in LE between at the mesometrial side and the anti-mesometrial side was similar. (**B**) Quantitative analysis of ABCA1 and SR-BI by measuring the average integrated optical density (IOD) in LE in Yorkshire pigs. Asterisks indicate significant differences (mean ± SD) between breeds (**P < 0.01), P value was determined by Student’s t test. Legend: Tr, trophoblast; LE, endometrial luminal epithelium; GE, glandular epithelium; D, day of pregnancy; Scale bars = 100 μm or 20 μm (amplification).

**Figure 3:**
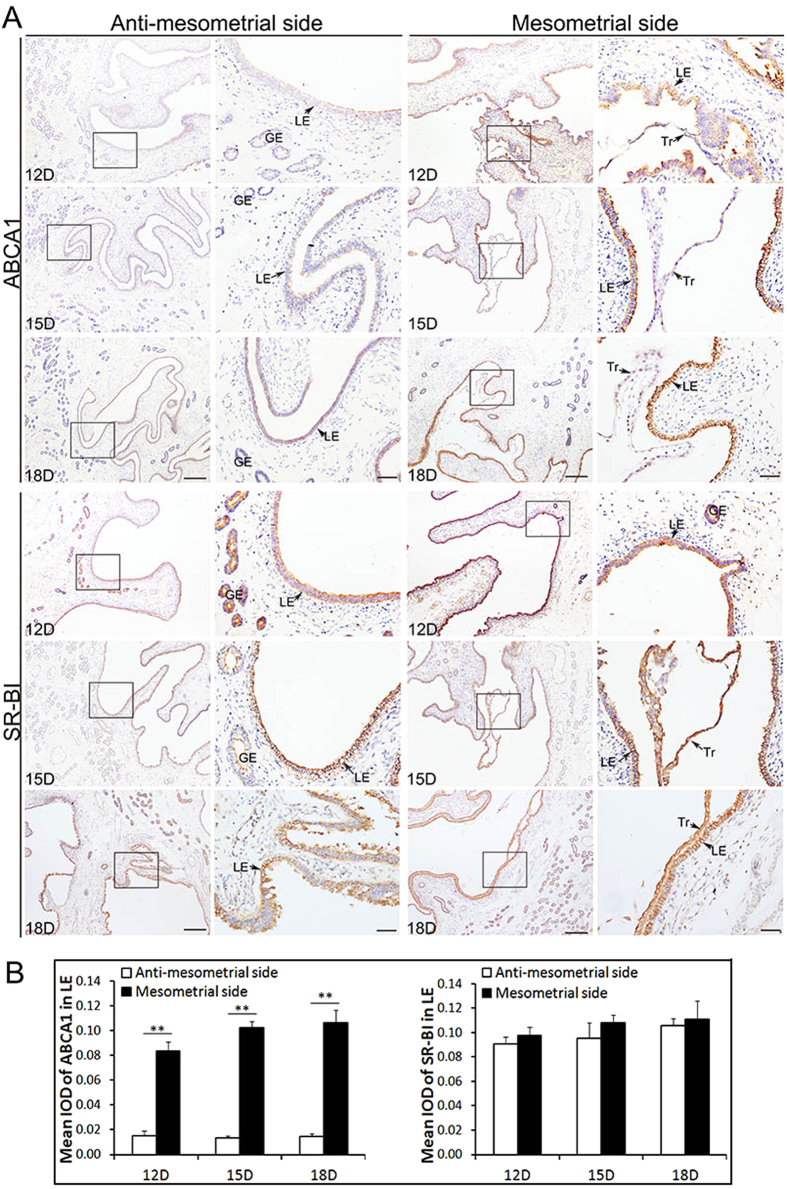
Immunohistochemical analysis of ABCA1 and SR-BI at the maternal-fetal interface (including the anti-mesometrial side and mesometrial side of the uterus) during the peri-implantation period in Meishan pigs. (**A**) Images stained with ABCA1 and SR-BI antibodies on Days 12, 15 and 18 of pregnency in Meishan pigs. The expression patterns of ABCA1 and SR-BI both in Meishan pigs and Yorkshire pigs were similar. (**B**) Quantitative analysis of ABCA1 and SR-BI by measuring the average integrated optical density (IOD) in LE. Asterisks indicate significant differences (mean ± SD) between breeds (**P < 0.01), P value was determined by Student’s t test. Legend: Tr, trophoblast; LE, endometrial luminal epithelium; GE, glandular epithelium; D, day of pregnancy; Scale bars = 100 μm or 20 μm (amplification).

**Figure 4:**
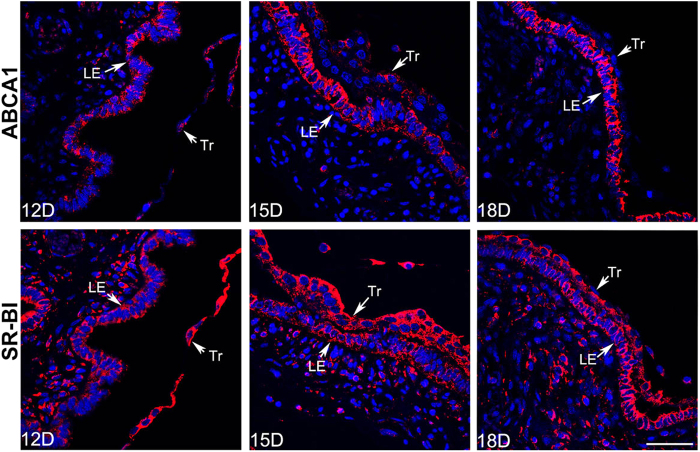
Confocal laser scanning microscopic localization of ABCA1 and SR-BI at the maternal-fetal interface (the mesometrial side of uterus) during the peri-implantation period in Yorkshire pigs. Positive signals of ABCA1 and SR-BI were shown in red and the blue staining represented nuclei (DAPI stained). The signals of ABCA1 and SR-BI were principally localized at the basolateral and apical side of the endometrial LE cell membrane. The sections stained with isotype matched normal rabbit IgG served as negative control (the control images of ABCA1 and SR-BI were in the Figs 6 or 8). Legend: Tr, trophoblast; LE, endometrial luminal epithelium; GE, glandular epithelium; D, day of pregnancy; Scale bar = 40 μm.

Schematic diagram Figure 1 shows that the attachment site of pig conceptus locates at the mesometrial side of uterine horn as demonstrated by previous studies^5^,^8^,^10^. (Fig. 1). The results of immunohistochemistry showed that ABCA1 was expressed in endometrial LE but barely detected on GE and Tr in Yorkshire and Meishan pigs (Figs 2 and 3). It is worth noting that the stain intensity of ABCA1 in endometrial LE at the mesometrial side was stronger than those at the anti-mesometrial side of uterus. The SR-BI protein was expressed primarily by the endometrial LE as well as GE and Tr in the two breeds (Figs 2 and 3). Contrary to the ABCA1, the stain intensity of SR-BI in LE between the mesometrial side and the anti-mesometrial side of uterus was similar. By comparison of the expression patterns of ABCA1 and SR-BI in Yorkshire and Meishan pigs, no difference was observed between the two pig breeds during the peri-implantation period. Subsequently, the laser confocal microscopy imaging was used to investigate the cell-specific localization of the two proteins. The ABCA1 was principally localized at the basolateral and apical side of the endometrial LE cell membrane (Fig. 4). Similarly, the localization of SR-BI was at the basolateral and apical side of the endometrial LE and Tr cell membranes (Fig. 4).

### Expression pattern of ABCA1 protein at the maternal-fetal interface in Yorkshire and Meishan pigs during the placenta development period (Days 26, 50 and 95 of pregnancy)

**Figure 5:**
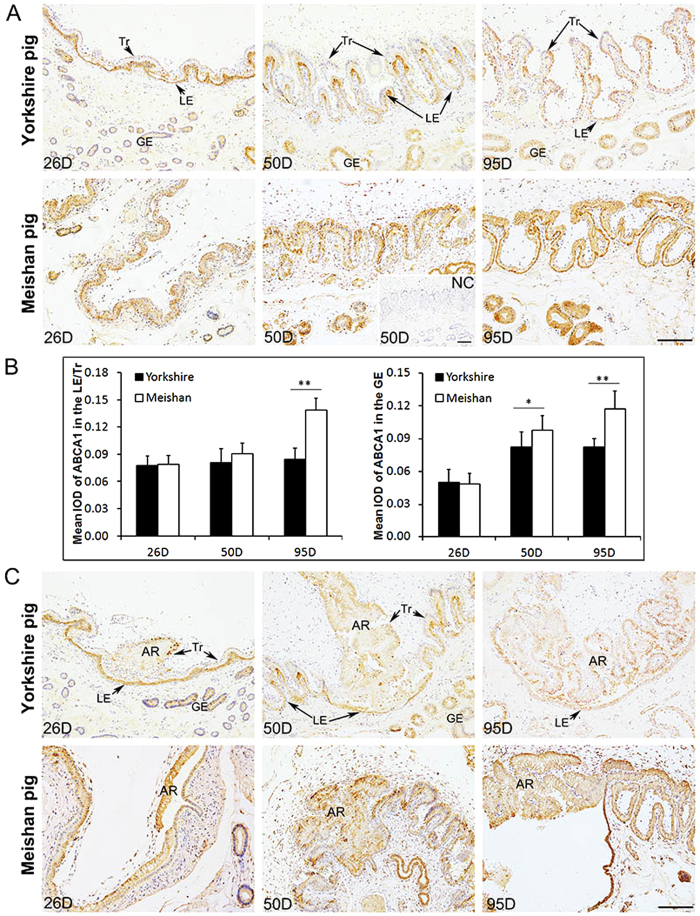
Immunohistochemical analysis of ABCA1 at the maternal-fetal interface and the placental areolae in Yorkshire and Meishan pigs. Uterine/placental sections were obtained on Days 26, 50 and 95 of pregnancy. Tissue sections were stained with rabbit anti-human ABCA1 polyclonal antibody.The sections stained with isotype matched normal rabbit IgG served as negative control. (**A**) Images were taken from the maternal-fetal interface in Yorkshire and Meishan pigs. ABCA1 positive cells were observed in the epithelial bilayer and GE in Yorkshire and Meishan pigs on Days 26, 50 and 95 of pregnancy. Legend: Tr, trophoblast; LE, endometrial luminal epithelium; GE, glandular epithelium; D, day of pregnancy; NC, negative control; Scale bar = 100 μm. (**B**) Quantitative analysis of ABCA1 by measuring the average integrated optical density (IOD) in the epithelial bilayer (composed by Tr and LE) and GE during pregnancy. Asterisks indicate significant differences (mean ± SD) between breeds (*P < 0.05; **P < 0.01. Analyzed by PROC MIXED of SAS). (**C**) Images were taken from the placental areolae in Yorkshire and Meishan pigs. The ABCA1 positive signals were filled in all the areolar regions, including Tr, LE and GE on Days 26, 50 and 95 of pregnancy. AR, areola; Scale bar = 100 μm.

**Figure 6:**
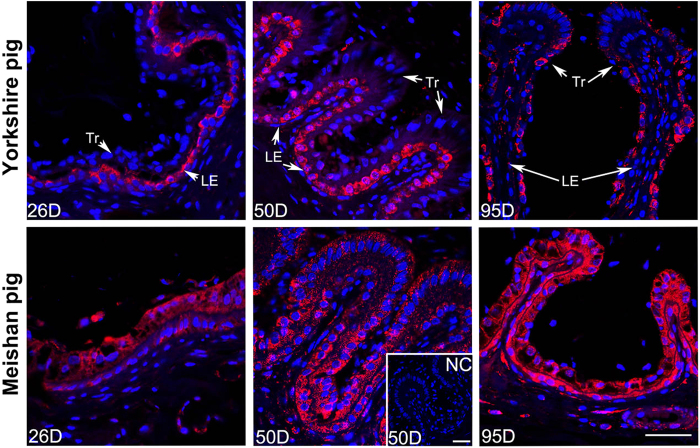
Confocal laser scanning microscopic localization of ABCA1 using rabbit anti-human ABCA1 polyclonal antibody at the maternal-fetal interface in Yorkshire and Meishan pigs. Positive staining of ABCA1 was shown in red and the staining was diffused in the positive cells (except the nuclei) without specific subcellular localization. The blue staining represents nuclei (DAPI stained). In Yorkshire pigs, the ABCA1 staining were mostly observed in LE on Days 26 and 50 of pregnancy, while on Day 95 of pregnancy, the positive signals were mostly observed in Tr and just a few positive signals could be observed in LE. In Meishan pigs, the ABCA1 staining was observed both in Tr and LE on Days 26, 50 and 95 of pregnancy. Legend: Tr, trophoblast; LE, endometrial luminal epithelium; GE, glandular epithelium; D, day of pregnancy; NC, negative control; Scale bar = 40 μm.

We found that the expression pattern of ABCA1 protein at the maternal-fetal interface was different between Yorkshire and Meishan pigs. In Yorkshire pigs, the ABCA1 staining was mostly observed in endometrial LE at all the 3 periods of pregnancy but was not detectable in Tr on Days 26 and 50 of pregnancy until on Day 95 of pregnancy. However in Meishan pigs, the ABCA1 staining was observed both in endometrial LE and Tr at the 3 periods of pregnancy (Fig. 5A). In addition, the results of quantitative analysis of ABCA1 expression at the epithelial bilayer (composed by Tr and endometrial LE) showed that the expression of ABCA1 at the epithelial bilayer was significantly higher in Meishan pigs than in Yorkshire pigs on Day 95 of pregnancy (Fig. 5B; P < 0.0001). And compared with the Yorkshire pigs, the staining intensity in the uterine GE was also significantly higher in Meishan pigs on Days 50 and 95 of pregnancy (Fig. 5B; Day 50, P = 0.0338; Day 95, P = 0.0001). The confocal results demonstrated that the positive staining of ABCA1 was diffused in the positive cells (except the nuclei) without specific subcellular localization in the two breeds (Fig. 6).

The maternal-fetal interface in pig placenta composes of two regions: the epithelial bilayer (interareolar region) and the areolar region. The pig placental areola is a special structure which locates at the openings of uterine gland and is responsible for uptake and transplacental transport of secretions from the uterine gland into the fetal circulation^42^,^43^. The immunofluorescence results showed that the ABCA1 was expressed in all the area of the placental areolae during pregnancy both in Yorkshire and Meishan pigs, and the staining intensity in the placental areolae was obviously higher in Meishan pigs although it was not quantified (Fig. 5C).

### Expression pattern of SR-BI protein at the maternal-fetal interface in Yorkshire and Meishan pigs during the placenta development period (Days 26, 50 and 95 of pregnancy)

**Figure 7:**
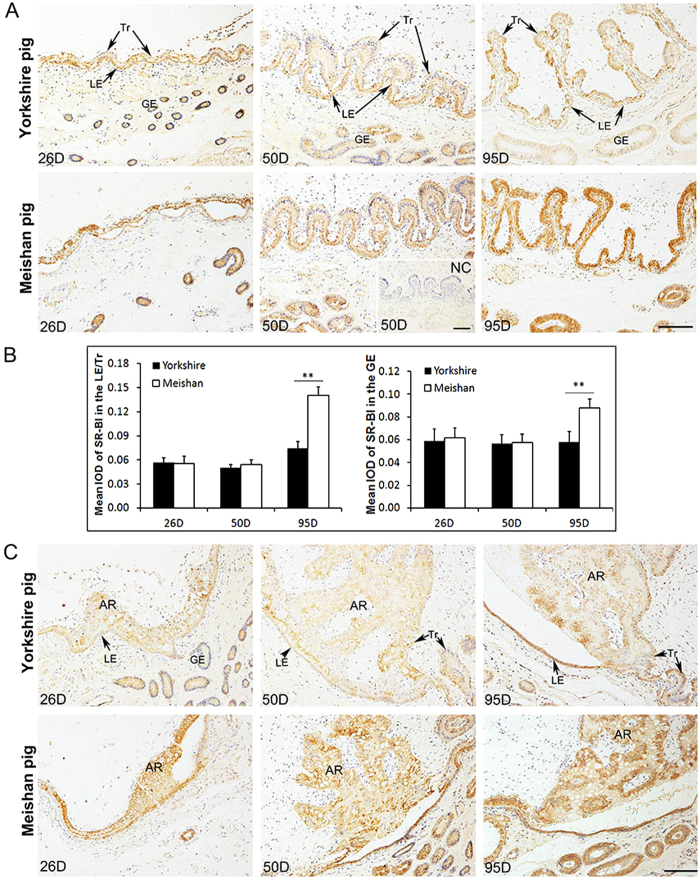
Immunohistochemical analysis of SR-BI at the maternal-fetal interface and the placental areolae in Yorkshire and Meishan pigs. Uterine/placental sections were obtained on Days 26, 50 and 95 of pregnancy. Tissue sections were stained with rabbit anti-human SR-BI polyclonal antibody. The sections stained with isotype matched normal rabbit IgG served as negative control. (**A**) Images were taken from the maternal-fetal interface in Yorkshire and Meishan pigs. SR-BI positive cells were observed in Tr, LE and GE in Yorkshire and Meishan pigs on Days 26, 50 and 95 of pregnancy. Legend: Tr, trophoblast; LE, endometrial luminal epithelium; GE, glandular epithelium; D, day of pregnancy; NC, negative control; Scale bar = 100 μm. (**B**) Quantitative analysis of SR-BI by measuring the average integrated optical density (IOD) in the epithelial bilayer (composed by Tr and LE) and GE during pregnancy. Asterisks indicate significant differences (mean ± SD) between breeds within a day (**P < 0.01. Analyzed by PROC MIXED of SAS). The data were shown as the mean ± SD. (**C**) Images were taken from the placental areolae in Yorkshire and Meishan pigs. The SR-BI positive signals were filled with all the areolar regions, including Tr, LE and GE on Days 26, 50 and 95 of pregnancy. AR, areola ; Scale bar = 100 μm.

Similarly to the ABCA1 expression patterns, the SR-BI was also expressed at the epithelial bilayer and uterine GE on Days 26, 50 and 95 of pregnancy in Meishan and Yorkshire pigs (Fig. 7A). Compared with the Yorkshire pigs, the immunostaining intensity of SR-BI at the epithelial bilayer and the uterine GE was also stronger in Meishan pigs on Day 95 of pregnancy (Fig. 7B; P < 0.0001), but was not observed significantly different between the two breeds on Days 26 and 50 of pregnancy. In addition, consistent with the ABCA1 expression pattern in the placental areolae, the SR-BI was also expressed in all the area of the placental areolae in the two breeds, and the staining intensity in the Meishan placental areolae was stronger (Fig. 7C).

**Figure 8:**
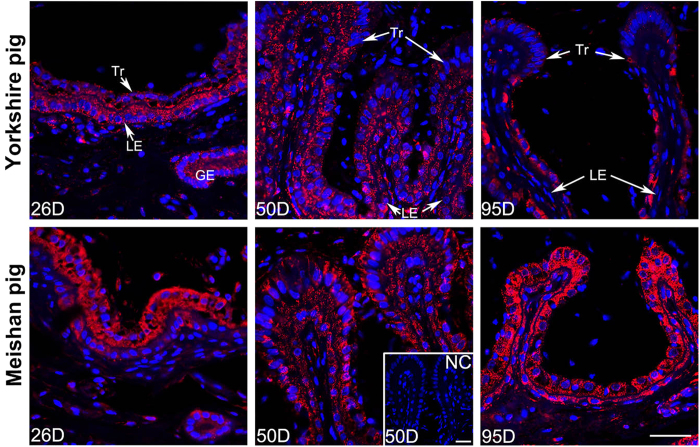
Confocal laser scanning microscopic localization of SR-BI at the maternal-fetal interface in Yorkshire and Meishan pigs. Positive staining of SR-BI is shown in red, blue staining represents nuclei (DAPI stained). The signals were mostly diffused in cells of Tr, LE and GE in Yorkshire and Meishan pigs on Days 26, 50 and 95 of pregnancy. And specially on Day 95 of pregnancy, in Yorkshire pigs, SR-BI staining were mostly observed in Tr and just few positive signals were observed in LE at the maternal-fetal interface; while in Meishan pigs, SR-BI signals were strongly located at the maternal-fetal interface, including all the Tr and LE. Legend: Tr, trophoblast; LE, endometrial luminal epithelium; GE, glandular epithelium; D, day of pregnancy; NC, negative control; Scale bar = 40 μm.

Furthermore, the results of confocal microscopy imaging indicated that the SR-BI staining signals were also mostly diffused in cytoplasm of the Tr, LE, and GE in Yorkshire and Meishan pigs on Days 26, 50 and 95 of pregnancy (Fig. 8).

### Expression of HMGCR mRNA in pig conceptuses/placentas

**Figure 9:**
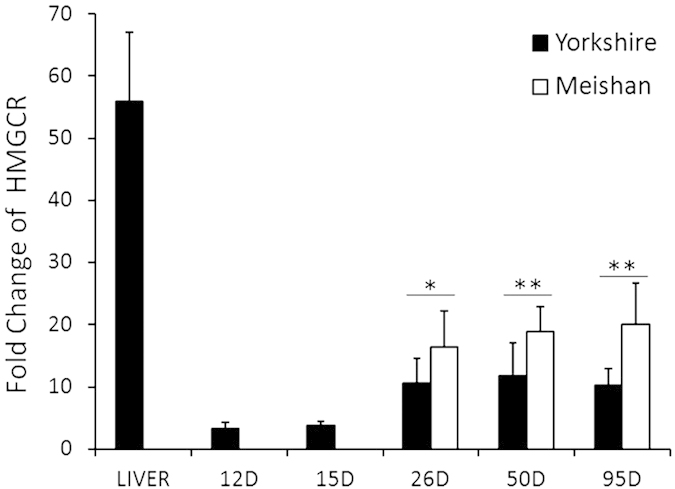
Quantitative RT-PCR was used to investigate the mRNA profiles of *HMGCR* in Yorkshire conceptus on Days 12 and 15 of pregnancy, in Meishan and Yorkshire placentas Days 26, 50 and 95 of pregnancy and in the glits livers. *HMGCR* mRNAs were highly expressed in the liver and placenta, but the levels of *HMGCR* mRNAs were very low in the conceptus. Asterisks indicate significant differences (mean ± SD) between breeds (*P < 0.05; **P < 0.01), P value was determined by Student’s t test.

As the HMGCR is the rate-limiting enzyme of cholesterol synthesis. In mammals, the liver is the most important site of the cholesterol synthesis, but some other types of cells can also produce cholesterol. By using the quantitative RT-PCR, we found that the HMGCR mRNA levels were higher in placentas (obtained from Days 26, 50 and 95 of pregnancy, respectively) but were very low in conceptuses (obtained from Days 12 and 15 of pregnancy, respectively). In addition, the HMGCR mRNA levels in Meishan placentas were significantly higher than those in Yorkshire placentas on Days 26, 50 and 95 of pregnancy (Fig. 9).

## Discussion

The present study determined the cellular location features of ABCA1 and SR-BI proteins in porcine Tr and endometrium during periods of peri-implantation and placentation. During the peri-implantation period in pigs (Days 12,15 and 18 of pregnancy ), the conceptus undergoes dramatic morphological change from spherical to tubular to filamentous forms^4^,^5^. Meanwhile, the conceptuses have the steroidogenic ability to synthesize and release estrogen. Estrogen is recognized as not only the signal for maternal recognition of pregnancy but also the stimulator for the secretion of the endometrium-derived histiotrophic nutrition for supporting the conceptus^6^,^7^. Due to the fact that cholesterols serves as the ultimate precursor for the biosynthesis of all steroid hormones including the estrogen, the cholesterol is critical for the conceptus development and successful implantation in pigs^21^,^22^. The 3-Hydroxy-3-methylglutaryl coenzyme A reductase (HMGCR) is the rate-limiting enzyme for cholesterol synthesis^44^,^45^. We found that the level of *HMGCR* mRNA was very low in the Yorkshire conceptuses during the peri-implantation period. It has been demonstrated that ABCA1 is a cell surface transporter that facilitates the export of cellular cholesterol and phospholipids to apolipoprotein A-I (apoA-I) to generate precursors for high-density lipoproteins (HDL) particles^30^. Consistently, our results showed that the ABCA1 protein was barely detected in conceptuses. These findings could suggest that the conceptuses may synthesize cholesterol at a very low rate and thereby mainly rely on the supply of the maternal-derived cholesterol. Consistent with the idea, we found that SR-BI, the receptor for HDL that mediates selective cholesteryl ester uptake and also the cellular cholesterol efflux, were detected to be expressed persistently on both apical and basolateral side of the conceptuse Tr, as well as endometrial LE and uterine GE form Days 12 to 18 of pregnancy. The expression patterns of the ABCA1 and SR-BI proteins in uteruses and conceptuses were similar between Yorkshire and Meishan pigs, but we failed to check the HMGCR mRNA levels in Meishan conceptuses due to poor quality RNA. Therefore, we speculated one possible model for cholesterol transfer from maternal circulation to the developing pig conceptuses during the peri-implantation period at least for Yorkshire pigs: (1) the cholesteryl esters could be taken up from the core of lipoproteins that are in the maternal circulation and released into uterine lumen through the receptor SR-BI expressed by the endometrial LE and uterine GE; (2) the receptor SR-BI expressed on the conceptuse Tr mediates the uptake of cholesterol into the fetal circulatory system.

The present study revealed an interesting finding that in contrast to SR-BI which exhibited uniform distribution on epithelium around the uterine lumen, ABCA1 was strongly expressed on epithelium at the mesometrial side of the uterus close to the implanting conceptus but showed very weak expression at the anti-mesometrial side. The significance of this observation is unclear. During the peri-implantation period, the floating pig conceptus is forced against the mesometrial area on which conceptus starts to implant by an orientating mechanism^8^. The uterine epithelium at area in close to or directly contacted by the trophoblast shows features that are in contrast to those of unapposed region, including changes in cell shape and secretion^46^. The intense local expression of ABCA1 implies the accumulation of cholesterol in uterine epithelium around the apposed region, where cholesterol acts as the cell structure component and activator of membrane-initiated signaling associated with the apposing and adhering of trophoblast to the epithelium. On the other hand, the contact area of endometrium by trophoblast is an immunologically privileged site where maternal immune system was tolerant to the implanting trophoblast. This is attributed to synchronous signaling between the conceptus and endometrium. Evidences showed that expression of ABCA1 could be regulated by several cytokines that are secreted by conceptus and endometrium, including interferon gamma, interleukin-1beta, interleukin-10 and transforming growth factor beta. In addition, ABCA1 also could modulate the secretion of cytokines, such as macrophage migration inhibitory factor^47^,^48^,^49^. Taken together, our findings may suggest that in addition to the activity in cholesterol export, ABCA1 may have a role in remodeling of the endometrium and modulating the local immunosuppression in the implanting time in pigs.

The pig epitheliochorial placentation is initiated from Days 15–20 of pregnancy^8^. Meanwhile, there is a gradual transition from only histiotrophic to mainly hemotrophic and histiotrophic nutrition. Around Days 26–30 of pregnancy, the pig placenta is established and becomes the main route of transport. Therefore, nutrients must move through either two sets of epithelial cell tight junctions (trophoblast and uterine luminal epithelial cells) or four sets of epithelial cell membranes and two cell cytoplasm to get from the maternal blood to the fetal blood in pigs. As the development of fetus and placenta progresses, the requirement of nutrients, including cholesterol, is increased. To enhance the efficiency of placental nutrients transporting, some exquisite changes of the placental structure are occurred in this period of pregnancy, including that: (1) the placental folds increase in size to increase maternal-fetal exchange surface area^17^,^50^; (2) the distance between maternal and fetal capillaries is decreased^51^. Besides the two physical factors, the observation that the expression levels of ABCA1 and SR-BI were increased as the pregnancy proceeds is in agreement with the idea that the nutrient specific transport mechanism is also of importance in placental function^5^. The ABCA1 and SR-BI were membrane transporters, and our confocal microscopy results showed that the ABCA1 and SR-BI were principally localized at the basolateral and apical side of the uterine LE cell membrane during the peri-implantation period, but from Days 26 to the end of pregnancy ABCA1 and SR-BI signals were diffused in the cytoplasm without any specific subcellular localization in Tr and LE in Yorkshire and Meishan pigs. Albrecht *et al.* reported the similar obsrvation in humans that ABCA1 was localized at the basolateral and apical side of cytotrophoblast cell membrane during early pregnancy, while ABCA1 signals were distributed among entire cell cytoplasm in term placentas^52^. Thus our results suggested that it may also exist the alternative mechanism for the maternal-fetal cholesterol transport in pigs by which the apolipoproteine and ABCA1 form complex on the cell surface and then the complex is internalized to late endosome to transport the lipid from cells by exocytosis during the placentation period^47^.

**Figure 10:**
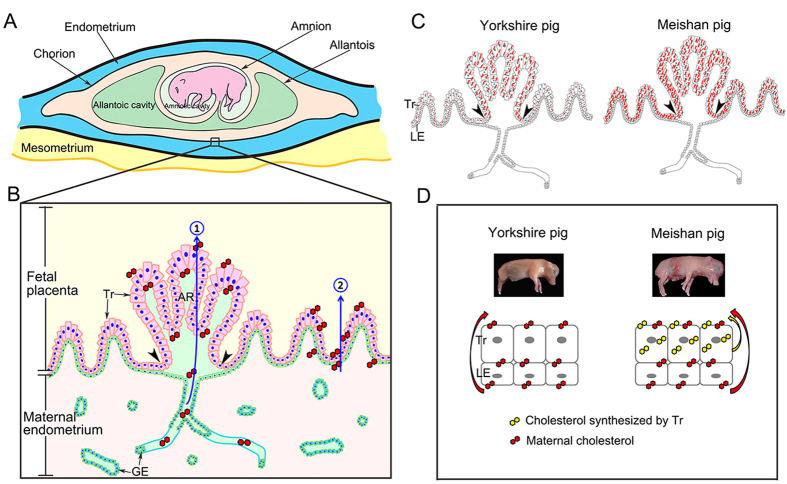
Illustration depicting the paths for cholesterol transport across the maternal-fetal interface in the pig placenta. (**A**) schematic drawing of the epitheliochorial placenta of pig. (**B**) the maternal-fetal interface of the pig placenta composed of two regions, the interareolar region (composed by trophoblast and luminal epithelium, outside of arrowheads) and the areolar region (located at the mouth of uterine glands, enclosed by arrowheads), respectively. Our findings revealed two paths of cholesterol transport from maternal to fetal circulation, 1 the areolar-gland subunit; 2 the placental epithelial bilayer; (**C**) Schematic illustration of ABCA1 expression patterns in placental trophoblast in Yorkshire and Meishan pigs, showing that the ABCA1 (in red) was barely expressed in trophoblast which located at the interareolar region (outside of arrowheads) in Yorkshire, but it was highly expressed in the trophoblast in Meishan pigs. (**D**) Schematic illustration of the proposed mechanism for Meishan pig to increase the placental efficiency in transport cholesterol. Legend: Tr, trophoblast; LE, endometrial luminal epithelium; GE, glandular epithelium; AR, areola of placenta.

In terms of the morphological characteristics, the pig maternal-fetal interface is composed of two specific regions: (1) trophoblast-endometrial epithelial bilayer (the interareolar region); and (2) the areolar-gland region where the specialized trophoblast cells locate at the mouth of uterine glands and the secretions from uterine glands are absorbed by placental areolae and then released into the fetal circulation^10^,^42^,^53^. This study revealed the differences of the ABCA1 and SR-BI in expression patterns and localization between Meishan and Yorkshire pigs in the two regions. First, although the SR-BI was expressed by the Tr in both the interareolar and areolar regions as well as the endometrial LE and uterine GE in the two pig breeds on Days 26, 50 and 95 of pregnancy, it was abundant on Tr in Meishan pigs than in Yorkshire pigs, especially on Day 95 of pregnancy. The finding suggests the role of SR-BI in cholesterol transport during the three stages of pregnancy. Second, ABCA1 was expressed by the endometrial LE in the two pig breeds on Days 26, 50 and 95 of pregnancy. In addition, different expression pattern of ABCA1 in Tr was observed between the two pig breeds. ABCA1 was abundant in both interareolar and areolar Tr in Meishan pigs. However, in Yorkshire pigs, the positive signals of ABCA1 were detected in areolar Tr during the three stages of pregnancy, but were barely detectable in interareolar Tr on Days 26 and 50 of pregnancy and intermittent on Day 95 of pregnancy (Fig. 10C). Interestingly, the mRNA levels of HMGCR, the rate-limiting enzyme for cholesterol synthesis, were significantly higher in Meishan placentas than those in Yorkshire placentas, suggesting that the Meishan placentas have the higher rate of cholesterol synthesis. This result is consistent with the report that compared to the Yorkshire pigs, the expression levels of most of the cholesterol synthesis genes in Meishan placentas were increased^54^. Moreover, the provided evidence also showed that compared with the Yorkshire pigs, the concentration of free and esterified cholesterol was higher in Meishan placentas from Day 45 to the end of pregnancy^55^. In contrast to SR-BI–mediated bidirectional cholesterol exchange (influx and efflux), efflux cholesterol via ABCA1 is unidirectional, occurring in the cells which are enriched with cholesterol^28^. Taken together, our data support the possibility that, in comparison to Yorkshire pigs, the higher expression level of ABCA1 in both interareolar and areolar Tr in Meishan pigs is associated with the increased rate of both the placental cholesterol biosynthesis and the maternally derived cholesterol uptake through SR-BI (Fig. 10D). As a consequence, the amount of cholesterol entering the Meishan fetal circulation could be increased in comparison to Yorkshire pigs at least through the ABCA1 mediated pathway. As compared to Yorkshire pigs, Meishan pigs maintain the smaller size of placenta so that more fetuses can be carried within the uterus^54^. The suggested compensatory mechanisms include that Meishan pigs have increased density of blood vessels^16^ and more complex placental folded bilayer^17^. Our findings could suggest another compensatory mechanism in response to the reduced placenta size for Meishan pigs by increasing the expression of ABCA1, one of the nutrient specific transporters.

In summary, we reported for the first time that the expression of cholesterol transporter ABCA1 was more intensive on endometrial epithelium in the mesometrial side of the uterus than the anti-mesometrial side during the peri-implantation period in pigs. The finding implies that the initiation of the accumulation of cholesterol mediated by ABCA1 in uterine epithelium was mesometrial, where pig conceptus attaches and mutual synchronous signaling between the conceptus and endometrium takes place as well during the period of implantation. Therefore we proposed that ABCA1 related pathway may be part of the orientating system involving mutual attraction between conceptus and the endometrial epithelium and thus is worth further investigation. In addition, we observed two paths for cholesterol transport from maternal to fetal circulation mediated by ABCA1 and SRBI, the areolar-gland subunit and the placental epithelial bilayer (Fig. 10B). Furthermore, the more expression levels of ABCA1 and *HMGCR* at interareolar and the areolae placental regions in Meishan pigs could imply that Meishan placenta has an increased efficiency of cholesterol transport to the developing fetus (Fig. 10D).

## Materials and Methods

### Animals and Tissue Collection

All researchs involving animals were conducted according to the Regulation of the Standing Committee of Hubei People’s Congress. All experimental protocols were approved by the Ethics Committee of Huazhong Agricultural University, P. R. China. Chinese Meishan and Yorkshire gilts were obtained from the breeding pig farm of Huazhong Agricultural University (Wuhan, China). Gilts were checked for estrus twice daily and mated naturally to boars of their own breed at the onset of estrus (Day 0) and again 12 h later. After the gilts were slaughtered at a local slaughterhouse on Days 12, 15, 18, 26, 50 and 95 of pregnancy (n = 3–4 gilts/breed/day of pregnancy). The uteri were removed rapidly and transported in an icebox to the laboratory. Pregnancy was confirmed by the presence of apparently normal filamentous conceptuses in uterine flushings on Days 12, 15 and 18 and presence of embryos and placenta on the later days of pregnancy. Then the cross-sections of uteri on Days 12, 15 and 18 of pregnancy were collected from three locations of each uterine horn: proximal (close to the fallopian tube), medial, and distal (close to the body of the uterus). On Days 26, 50 and 95 of pregnancy, the uteri were opened longitudinally along the anti-mesometrial side, and the cross-sections of the uteroplacental interface (including myometrium, endometrium, and placenta) in the implantation sites were collected from three locations of each uterine horn: proximal, medial, and distal. These sections samples were fixed immediately in fresh 4% paraformaldehyde in PBS (pH 7.2) for 24 h followed by paraffin embedding for HE staining, immunohistochemistry and confocal microscopy. The conceptuses on Days 12 and 15 of pregnancy and the placental tissues on the later days of pregnancy were collected, frozen in liquid nitrogen, and stored at −80 °C for RNA extraction.

### RNA extraction and cDNA synthesis

Total RNA was isolated from the conceptuses and the placental tissues using Trizol reagent (Invitrogen, Carlsbad, CA, USA) following the manufacturer’s instructions. The integrity of RNA was determined by electrophoresis in 1% agarose gel. The quantity and purity of RNA were determined using a NanoDrop™ 2000 spectrophotometer (Thermo Scientific). Genomic DNA elimination and cDNA synthesis were conducted using PrimeScript RT Reagent Kit with genomic DNA (gDNA) Eraser (Perfect Real Time, TaKaRa) according to the manual. For each sample, 1 μg of total RNA was used for each 20 μL reverse transcription reaction system. All cDNA samples were preserved at −20 °C.

### Quantitative RT-PCR Analysis

To analyze levels of *HMGCR* mRNAs in the conceptuses and placental tissues, real-time RT-PCR was carried out on the CFX384 Real-Time PCR Detection System (Bio-Rad) using the SYBR Green method. The PCR reaction volume was 10 μL consisting of 2 ng/μL cDNA, 0.3 μM primers, 2 × SYBR Green qPCR Master Mix (Toyobo co., ltd, Osaka, Japan), and RNase- and DNase-free sterile water. PCR conditions were as follows: single cycle of 5 min at 95 °C, followed by 40 cycles of 30 sec at 95 °C, 20 sec at 62 °C, and 15 sec at 72 °C. The primers were designed by Oligo 7 Primer Analysis Software (Molecular Biology Insights, Inc.). The primer sequences and product size were listed in Table 1. The GAPDH gene was used as a control. The PCR amplifications were performed in triplicate for each sample.

### Immunohistochemistry

To determine ABCA1 and SR-BI expressions at the uterine/placental cells from different pig breeds during pregnancy, immunohistochemical analysis was performed. Sections (4 μm thick) were deparaffinized with xylene and rehydrated in an alcohol gradient, 100% ethanol, 95% ethanol, 90% ethanol, 80% ethanol, 70% ethanol, and distilled water. Then tissue sections were treated with 3% hydrogen peroxide (H_2_O_2_) to block endogenous peroxidase for 15 min at room temperature. After rinsing with distilled water, sections were submitted to heat-induced epitope retrieval by microwave treatment in 0.01 M sodium citrate buffer (pH 6.0) in a microwave oven at 750 W for 15 min (three times for 5 min each). Afterwards, the sections were cooled for 30 min at room temperature and rinsed three times in phosphate-buffered saline (PBS), 5 min each. The sections were blocked with 5% bovine serum albumin (BSA) in PBS for 30 min in a humid chamber at room temperature. The sections were incubated with a rabbit anti-Human ABCA1 polyclonal antibody (1:300, Novus, NB400-105) or with a rabbit anti-Human SR-BI polyclonal antibody (1:300, Novus, NB400-104) at 4 °C overnight and then in biotinylated goat anti-rabbit secondary antibody (1:100, SA1022, Boster Corporation, China). Following immunostaining, sections were counterstained with hematoxylin and mounted. For each sample, a negative control was performed by replacing the primary antibody with corresponding nonspecific immunoglobulin G (IgG). All sections were stained immunohistochemistry under the same conditions.

Images of the immunohistochemical staining were taken by an olympus microscope BX-53 and digital camera DP26 (Olympus, Japan) respectively. For each gilt, 3 healthy uterine/placental units were studied as individual samples. Immunofluorescence and immunohistochemical staining was analyzed by mean integrated optical density (IOD) using ImagePro Plus 6.0 software (Media Cybernetics, Silver Spring, USA) as described by Hong *et al.*^18^. Photographic plates were assembled using Adobe Photoshop CS6 (Adobe Systems Inc.).

### Immunofluorescence and confocal scanning laser microscopy

To determine the cell-specific localization of the ABCA1 and SR-BI proteins in the porcine uterine/placental cells during pregnancy, confocal laser scanning immuofluorescence microscopy was used. Rabbit anti-Human ABCA1 polyclonal antibody (1:300, Novus, NB400-105) and rabbit anti-Human SR-BI polyclonal antibody (1:300, Novus, NB400-104) were used in immunofluorescence staining. The sections were processed as immunohistochemistry until reaching the step of incubating secondary antibodies. For incubating secondary antibodies, the sections were incubated with fluorescence-labeled secondary antibodies (1:100, BA1032, Boster Corporation, China) at 37 °C for 30 min. The nuclei were counterstained with 4**′**, 6-diamidino-2-phenylindol (DAPI). For each sample, a negative control was performed by replacing the primary antibody with corresponding nonspecific immunoglobulin G (IgG). All sections were stained immunofluorescence under the same conditions. After mounting, the slides were watched under an LSM 510 META laser confocal microscope (Carl Zeiss, Jena, Germany). The data were processed by using the the LSM 510 META (Zeiss) software, and images were assembled by using Adobe Photoshop CS6 (Adobe Systems Inc.).

### Statistical Analysis

All data were shown as means ± SD. The date of the quantitative RT-PCR for the HMGCR mRNA were evaluated using Student’s t-Test. The date of immunoreaction intensity for ABCA1 and SR-BI were analyzed using PROC MIXED (Version 8.1; SAS Institute, Inc., Cary, NC), using a model that included the fixed effects of day of pregnancy (26, 50 and 95), breed (Yorkshire and Meishan), and the breed × day interaction. Gilt within breed × day was included as a random effect. A significance level of P < 0.05 was considered significant.

## Additional Information

**How to cite this article**: Hong, L. *et al.* Difference in expression patterns of placental cholesterol transporters, ABCA1 and SR-BI, in Meishan and Yorkshire pigs with different placental efficiency. *Sci. Rep.* **6**, 20503; doi: 10.1038/srep20503 (2016).

## Figures and Tables

**Table 1:** Summary of PCR primer sequences and expected product sizes.

Gene Name	Primer sequences (5′-3′)	Product Size (bp)	GeneBank Accession No.
*HMGCR*	CACATTCACCCTCGATGCTCT	165	NM_001122988.1
	TCTAAAACCAAGGACACGCAAG		
*GAPDH*	CACCAGGGCTGCTTTTAACTCTG	154	NM_001206359.1
	GATGACAAGCTTCCCGTTCTCC		
